# Influence of Healing Abutment Height on Secondary Implant Stability Using Resonance Frequency Analysis: A Prospective Clinical Study

**DOI:** 10.3390/jcm14145140

**Published:** 2025-07-19

**Authors:** Alicia Martín-Martín, Esteban Pérez-Pevida, Saray Férnandez-Hernández, Jaime Lubillo-Valdeón, Aritza Brizuela-Velasco

**Affiliations:** 1 Department of Surgery, Faculty of Medicine, University of Salamanca, C/Alfonso X El Sabio s/n, 37007 Salamanca, Spain; atmartin@usal.es; 2 Faculty of Oral Sciences, Miguel de Cervantes European University, C/del Padre Julio Chevalier 2, 47012 Valladolid, Spain; sfernandezh@uemc.es (S.F.-H.); jlubillo@uemc.es (J.L.-V.); 3 DENS-iA Research Group, Faculty of Health Sciences, Miguel de Cervantes European University, C/del Padre Julio Chevalier 2, 47012 Valladolid, Spain; abrizuela@uemc.es

**Keywords:** implant stability, resonance frequency analysis, healing abutments, one surgical phase, insertion torque, marginal bone level

## Abstract

**Background/Objectives:** The aim of the present study is to evaluate the influence of the healing abutment height on secondary implant stability measured by resonance frequency analysis. In this prospective observational clinical study of 30 implants, the secondary stability of the implant was measured via resonance frequency analysis of the abutment during the osseointegration process. **Methods:** Two groups were compared: a <4 group (*n* = 15), with a space between the healing abutment and the antagonist of <4 mm, and a ≥4 group (*n* = 15), with a space of ≥4 mm. **Results:** Statistically significant differences (*p* < 0.05) in the implant stability values obtained at surgery (T0) and at the eighth week of osseointegration (T8) were observed between the two groups, with higher values for the <4 group. Pearson’s correlation analysis revealed a trend towards a significant relationship with the mean force (−0.6546) and a linear inverse relationship, so that by decreasing the distance between the abutment and the contact with the antagonist, the secondary implant stability values increased. A comparison of the mesial and distal peri-implant marginal bone levels at T0 and T8 did not reveal statistically significant differences (*p* > 0.05). A greater healing abutment height, placing it closer to the antagonist, increases and accelerates secondary stability, as measured by resonance frequency analysis. **Conclusions:** The results of the study support the recommendation of using high healing abutments, placing the abutment close to the opposing occlusal plane, according to biomechanical criteria.

## 1. Introduction

Dental implant osseointegration, as a biological event, has usually been equated with a model of primary or direct healing of a bone fracture; in both cases, mechanical stability and a favorable biological environment are necessary for the success of the process [1]. Several studies in animal models have revealed the biological processes that take place at the histological level until the apposition of bone directly on the implant surface, which characterizes osseointegration. Ostectomy of the maxillary bone, the aim of which is to surgically create the implant bed, immediately results in the activation of hemostasis, which is initially governed by platelet aggregation. Specifically, subsequent platelet degradation plays an essential role in the activation of the second phase of the process, which is inflammation [2]. This phase is maintained until the third or fourth day; the area of the bone–implant interface is occupied by inflammatory cells, red blood cells, and cellular degradation elements, maintaining space that will later be used during angiogenesis. The resulting neovascularization marks the beginning of the third phase, the proliferative phase, during which osteoblastic cells are recruited to the interface and end up anchored in the titanium of the implant by means of integrins previously adsorbed in a nonspecific way on the implant surface. After two weeks, a reticular structure of poorly oriented three-dimensional bone tissue begins to be observed but is attached to the titanium surface of the implant; this structure will subsequently undergo a process of progressive calcification of its extracellular matrix until the eighth or tenth week [3,4].

To ensure successful healing of a fracture, mechanical control at the edges of the fracture is essential, and for this reason, the classic treatment for fractures is bone splinting. Various studies in animal models have shown that a lack of mechanical stability at this level leads to the rupture of the newly formed capillaries during the proliferative phase, and if this mechanical instability is maintained over time, repair by fibrous tissue is foreseeable [5]. This same phenomenon can occur during the osseointegration of an implant so that, to avoid it, ensuring implant stability is necessary. Some authors define implant stability as the absence of clinical mobility of the implant. However, other studies have shown that micromovements ranging from 50 to 150 microns are tolerable and do not impair the process or maintenance of osseointegration [6]. Therefore, it would be more correct to define implant stability as the control of micromotion at the bone–support–implant interface. Primary stability, for its part, is achieved at the time of implant placement in the supporting bone; primary stability is purely mechanical in nature and highly influenced by factors such as bone density, instrumentation, and implant design, among other factors. Several studies have revealed that this stability decreases during the first weeks of the osseointegration process to be progressively replaced by secondary stability, which is essentially biological and that takes place as a result of the apposition of the osteoid matrix and its subsequent calcification, as previously described [7,8].

The assessment of osseointegration can be carried out through various methods, some of which lack possibilities for clinical application because they are invasive, such as histomorphometric studies for the assessment of bone-to-implant contact (BIC) or disinsertion torques, both of which are commonly applied in animal model studies [9]. Other methods, however, can be used in clinical applications, such as the evaluation of marginal bone levels and implant stability [10].

In this context, monitoring the evolution of peri-implant marginal bone levels during osseointegration is carried out mainly through the analysis of two-dimensional radiographs. For this purpose, the most common method is periapical intraoral radiography with a parallelizer. The objective is not only to obtain periapical radiolucent images of the implant body that allow assessments to determine the integration stage but also to measure, by comparing different phases of the osseointegration process, the mesial and distal marginal bone levels. For this method to be useful, standardization is needed, as it will allow successive intraoral radiographs to be compared efficiently during the analysis; however, this method has the fundamental limitation that it allows assessment of only proximal marginal bone levels, and key areas such as the vestibular cortical bone are not analyzable because they are hidden by the radiopacity of the implant [11,12].

Although various methods have been used to measure implant stability, the two methods that are most supported by evidence are insertion torque measurement and resonance frequency analysis (RFA). The main limitation of insertion torque measurement is that it can be applied only for monitoring primary stability. However, RFA has no limitations in this regard. In RFA, an electromagnetic transducer and a machined abutment at the implant connection are employed, allowing the natural frequency of vibration of the implant within the supporting bone to be evaluated. The values are expressed as an implant stability quotient (ISQ), where 0 is the lowest possible stability and 99 is the highest [13]. Various studies have shown that the ISQ is correlated with the likelihood of achieving and maintaining osseointegration of the implant. A range of clinical ISQ values (from 55 to 80) are reported in the scientific literature, with an average value of 69 in the various maxillary locations after one year of loading. Primary stability ISQ values below 40 are considered to indicate a high likelihood of osseointegration failure, whereas ISQ values above 69 are considered to indicate a high likelihood of success [14]. However, the ISQ values are correlated with the expected micromovement at the bone-implant interface before the application of load [15] and with the stiffness of the supporting bone [16]. Measurement of the ISQ throughout the osseointegration process reveals the stabilization processes that are taking place, since only the mass and elasticity (which in the end are the factors that determine the natural frequency of vibration) of the bone (i.e., not of the implant) are predicted to change. On the other hand, it is necessary to take into account that both methods express totally different inertias: the insertion torque is a measurement of the mechanical resistance to apical advancement of the implant rotating around its longitudinal axis as a result of friction against the bone bed, whereas the ISQ is based on the strength of the contact of the implant with its bed and the resistance of the implant to lateral displacement [17].

Nevertheless, the two measurement methods can be used to confirm the primary stability of the implant and make clinical decisions such as whether to perform surgery in one or two stages or the loading protocol, including the immediate phase, which involves the connection of the prosthesis within the first week of implant placement [18]. In this sense, immediate loading has a series of obvious aesthetic and phonetic advantages for the patient, although with limited chewing functionality; however, immediate loading also has implications in implant osseointegration and stability due to the load transfer to the peri-implant bone in a scarring process. For example, in the 2019 retrospective observational clinical study by Brizuela and Chávarri [1], when a sample of implants rehabilitated with immediate loading was compared with another sample with conventional loading, the greatest increase in the implant stability values occurred in both groups at the time of implant loading—that is, immediately in the group subjected to immediate loading and after osseointegration in the group subjected to conventional loading. Additionally, there were no statistically significant differences in the success of osseointegration between the groups. This observation led us to hypothesize that immediate loading could have not only beneficial histological effects on the osseointegration of the implant but also a certain effect on adaptive remodeling to the load among implants integrated under this protocol; however, the study could not corroborate this finding because of the unconventional nature of the trial itself. The same research group, in an experimental study of an animal model (New Zealand breed rabbits) [19], placed implants for human use in tibias for their integration, establishing two groups: a control group of animals housed during the process and another case group of rabbits that ran on a specially adapted treadmill for one hour a day during the month of osseointegration. Compared with the control group, the case group presented a greater increase in the RFA secondary ISQ values but also a greater percentage of BIC and coronal cortical bone overgrowth. In view of these results, the authors concluded that the conventional loading of dental implants, which requires their complete integration in a period of between 8 and 12 weeks and has its origin in the initial protocols established by Bränemark, reflected a belief that did not align with biological observations.

Immediate loading has a fundamental limitation—cost—since it requires the development of two prostheses, a provisional one during the osseointegration period and a definitive one for the definitive load. This circumstance could be overcome through the selection of a raised healing abutment, which, as it is closer to the opposing occlusal plane, is more influenced by chewing forces during integration, and more load is transferred to peri-implant bone. The choice of the height of the healing abutment, in clinical practice, is not based on evidence. On the one hand, a closure cap is used when surgery is intended to be performed in two stages, as after the osseointegration period, an implant discovery surgery is necessary. However, when a one-stage surgery is performed, it is generally possible for clinicians to choose different types of healing abutments of different heights and geometries subjectively [20].

Therefore, this study aims to determine whether a healing abutment with a separation of less than 4 mm or greater than or equal to 4 mm with respect to the antagonist occlusal plane influences dependent variables related to osseointegration, such as secondary ISQ values measured by RFA and the level of peri-implant marginal bone measured by periapical radiography.

## 2. Materials and Methods

To meet the objectives set, an analytical and prospective observational clinical study that can be considered a cohort study was designed. The recommendations of the Strengthening the Reporting of Observational Studies in Epidemiology (STROBE) statement were followed. The recruitment was carried out among patients at the Dental Clinic of the Miguel de Cervantes European University who were likely to receive an implant to retain a crown that replaced a lost tooth. The surgical and rehabilitation procedures were carried out by the faculty team of the permanent training Master Pro-IR: Periodontology, Implantology, Oral Rehabilitation and Regeneration, from the same university. Sample recruitment took place from October 2023 to September 2024. The period of evaluation of the relationship between the exposure and the effect was the period of implant integration, which was standardized to 8 weeks in all cases for a protocol of conventional load, as defined by the consensus collected in the literature [18]. This study was submitted for evaluation by the Miguel de Cervantes European University Ethics Committee and approved in March 2023 (C.E.I. 5/2023). All patients received specific information about the study and signed an informed consent form.

### 2.1. Inclusion Criteria

- Men and women at least 18 years of age and in good general health.
- Absence of a single tooth in FDI positions 4, 5, 6, or 7 (premolar or molar region) requiring replacement with a single-unit implant.
- Request by the patient for a dental implant rehabilitation in the edentulous area.
- Commitment by the patient to attend the control visits throughout the duration of the study.

### 2.2. Exclusion Criteria

- Patients classified as class III or IV of the classification of the American Society of Anesthesiologists (ASA).
- Patients receiving bisphosphonates.
- Patients who smoked more than 10 cigarettes/day.
- Situations in which the teeth in the treated location were extracted at least 3 months before the day of the implant placement surgery and the alveolar ridges were incompletely healed.
- Patients with prior guided bone regeneration treatment in the area to be treated within the last 6 months.
- Absence of a minimum width of 1.5 mm in the contour of the implant and a minimum height of 2 mm to the lower dental nerve to allow the placement of conventional-size Klockner Essential Cone^®^ implants (Soadco, Escaldes-Engordany, Andorra) with polished necks of 1.5 mm; internal connections of 4.0 or 4.5 mm in diameter; and 8, 10, or 12 mm in length.
- Edentulous antagonist arch or rehabilitated with a partially or completely removable prosthesis.
- Implant insertion torque less than 15 Ncm
- Presence of signs of periodontal disease activity, such as suppuration and plaque index and bleeding less than 15%, according to the Mombelli criteria [21]

**Figure 1:**
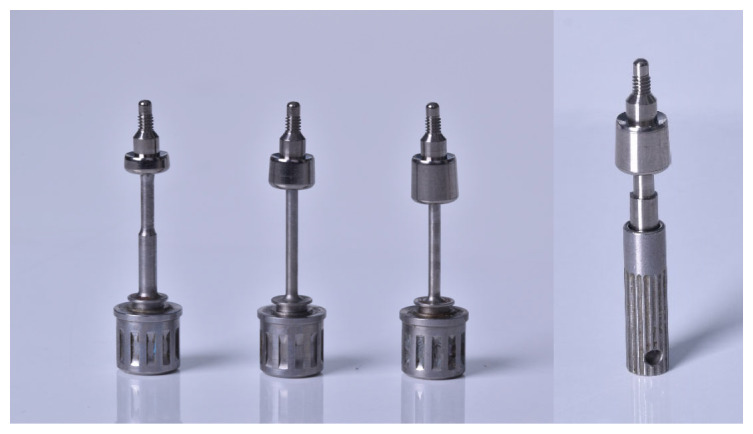
(**Left**), healing abutments used in the study, with heights of 1, 3, and 5 mm, on the corresponding screwdriver. (**Right**), 5 mm healing abutment with a screwed multipeg transducer and Penguin^®^ screwdriver.

The transepithelial healing abutments used were made of Grade IV titanium and had heights of 1, 3, and 5 mm but with identical geometry and support on the polished neck platform of the Essential Cone^®^ implant (Figure 1). Additionally, these abutments had a special device in their coronal area that allowed the monitoring of stability by means of RFA—a Penguin^®^ multipeg transducer (Integration diagnostics Sweden ab, Goteborg, Sweden) (Figure 1), thus allowing the assessment of the evolution of stability in the different phases of follow-up without the need to unscrew the healing abutment from the implant connection.

### 2.3. Study Variables

- The main independent variable was the separation of the coronal part of the healing abutments in contact with the opposing arch (mm). Two fundamental study groups were generated: those with a separation of less than 4 mm (<4 group) and those with a separation equal to or greater than 4 mm (≥4 group).
- The main dependent variable was the evolution of implant stability during the osseointegration process and the secondary ISQ values according to RFA.
- The secondary dependent variable was the evolution of the peri-implant marginal bone levels according to parallelized radiography at surgery and at the end of the osseointegration process (mm).

The main objective of several of the exclusion criteria, such as the number of cigarettes smoked, the state of healing or bone availability, and the minimum insertion torque, was to eliminate covariates that could influence the osseointegration process and thereby make establishing the relationship between the exposure and effect of our study difficult.

### 2.4. Surgical Protocol and Measurement of Variables at T0

The edentulous area to be treated was anesthetized by articaine infiltrative anesthesia with 40/0.01 mg/mL epinephrine.

A crestal incision was made in the edentulous area, detaching a full-thickness flap but without vertical discharge. The drilling sequence recommended by the implant manufacturer (Soadco, Escaldes-Engordany, Andorra) was used for each patient. The implant was placed using a torque wrench, taking into consideration that the apical limit of the polished neck of the implant would be in a juxtaosseous position.

Although the insertion torque was measured and annotated, it was of little interest, except to ensure that it exceeded the 15 Ncm mentioned in the exclusion criteria. This cutoff was taken into account to avoid complications when screwing and unscrewing the RFA multipeg transducers, which, according to the manufacturer’s instructions, must be manually tightened (<10 Ncm).

The allocation of the exposure (independent variable = height of the transepithelial healing abutment) was determined for each case once the implant placement was completed, taking into account the particular conditions of the same and considering that the healing abutment, once repositioned and when sutured, should be at a minimum of 1 mm supragingival height, and in no case should it have occlusal contact with the antagonist or be less than 0.2 mm from the occlusal plane. Taking these circumstances into account, a 1, 3, or 5 mm abutment was chosen.

**Figure 2:**
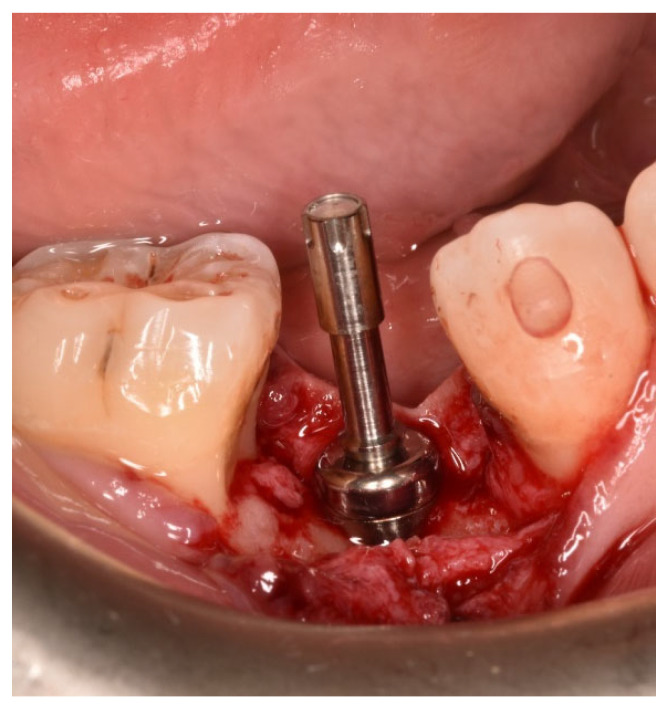
Implant stability quotient (ISQ) measurement with the transducer to the selected healing abutment.

For each patient, the selected abutment was screwed to the implant connection with a preload of 10 Ncm, and the ISQ value was subsequently measured via RFA by manually screwing the multipeg onto the abutment (T0) (Figure 2).

The flap was repositioned by simple suture, and written guidelines for postsurgical care were given to all patients: maintenance of antibiotic prophylaxis for 7 days, an anti-inflammatory regimen for the first 48 h, and application of local 0.12% chlorhexidine gel after meals.

**Figure 3:**
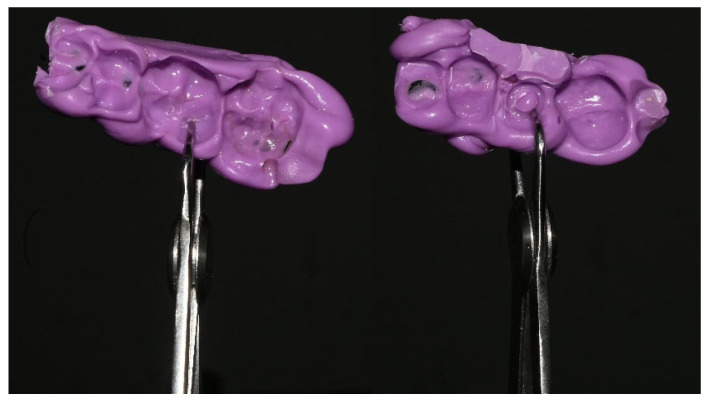
Iwanson calipers used to measure interocclusal spaces. On the (**right**), the area where the imprint of the healing abutment is marked is measured, and on the (**left**), the gauge positioned on the other side of the bite model on the occlusal side of the antagonist is shown.

After the intervention, two additional measurements were performed:

Oclufast Rock^®^ (Zhermack^®^, Rovigo, Italy) was used to generate a sectorial bite model of the abutment and antagonist area, ensuring that the patient occluded at maximum intercuspation. The model was slightly perforated in the contact areas between the teeth and their antagonists, ensuring that the material did not occupy a space that could modify the vertical dimension, and once performed, the interocclusal space was measured outside the mouth using an Iwanson caliper, positioning it on the coronal surface of the abutment and opposing tooth; this measurement corresponds to the separation of the abutment with respect to the opposing tooth (Figure 3).

**Figure 4:**
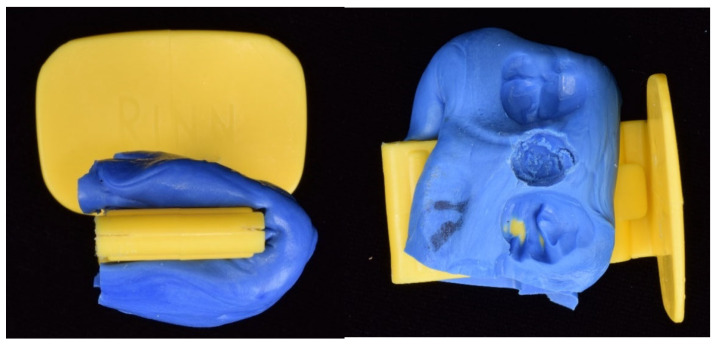
Silicone keys were made to adapt to the parallelizer positioner at the same position in weeks T0 and T8. In the image on the (**left**), you can see how the key is open on one side to be able to remove it and reposition it, and in the image on the (**right**), you can see how the key is marked with the two teeth adjacent to the implant and the footprint of the healing abutment.

Finally, intraoral radiography (T0) was performed in parallel technique with a positioner (XCP RINN; Dentsply Sirona, Charlotte, NC, USA), which was additionally individualized by means of a fingerprint of the opposing piece generated with the addition of heavy silicone (3M ESPE, Seefeld, Germany), which ensured the same projection when repeating the radiograph at T8 (Figure 4).

The suture was removed 15 days after the intervention. Throughout the duration of the study, the patients had to report any change in their health status, as well as the appearance of any complications such as pain, paresthesia or peri-implant infection, all of which had to be recorded in the corresponding control sheet.

Antibiotic administration was instituted with amoxicillin 750 mg/8 h or clindamycin capsules of 300 mg/8 h in patients allergic to penicillin for 7 days and anti-inflammatory agents (ibuprofen 600 mg/8 h) or analgesics (paracetamol 1 g/8 h) in allergic patients or with contraindications to the use of ibuprofen depending on the degree of pain.

### 2.5. Follow-Up Measurements

The data collection times for each dependent variable were as follows:

- T0: implant placement, RFA primary stability measurement, and parallelized radiography.
- T2: suture removal, 2 weeks after surgery, RFA stability measurement.
- T4: control visit at 4 weeks after surgery, RFA stability measurement.
- T8: at 8 weeks, after the osseointegration period, RFA secondary stability measurement and parallelized radiography.

**Figure 5:**
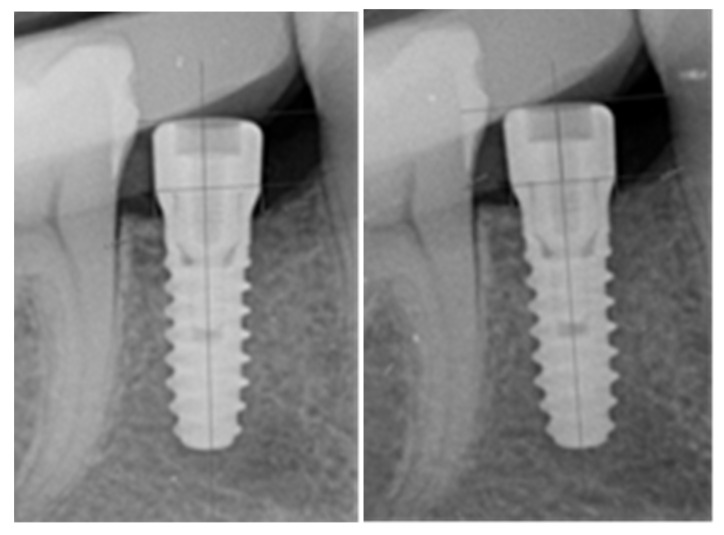
Periapical X-rays at weeks 0 (T0) and 8 (T8), performed with a silicone key and parallelization technique.

A comparison of the evolution of the marginal bone levels during the follow-up (T0 to T8) (Figure 5) was performed using the Vista Scan MiniView 2.0 software R1.3 version (Dürr Dental, Stuttgart-Feuerbach, Germany) and its measurement tool, expressed in tenths of mm. For this purpose, a fixed point was taken as a reference in the mesial and distal areas of the implant, which was the transition between the polished neck and the treated surface of the implant, an easily observable limit in the radiograph. From this point, the distance to the most coronal portion of the bone crest in contact with the implant was measured, both mesially and distally, on the day of surgery (T0) and at the end of osseointegration (T8). The main surgeon (AMM) was in charge of performing all the measurements during the development of the study. In parallel, a second surgeon, who was blinded to the results of the main surgeon, repeated the measurements of all the samples to determine the reproducibility of the determination. Lin’s concordance correlation coefficient analysis revealed good concordance between surgeons (0.6999). The final values recorded corresponded to those of the main surgeon.

### 2.6. Statistical Analysis

The variables used in this study are presented as their means, medians and standard deviations. Before the application of the statistical inference tests, the normality and homoscedasticity of the variables were verified. The verification of normality was performed with the help of the Anderson–Darling test, whereas homoscedasticity was verified using the F test for the case of normal distributions and the Levene test for the remaining distributions.

In the case of normal and homoscedastic variables, Student’s t test was used to determine if there were statistically significant differences between the means of the two categories under study. For the normal variables, but not the homoscedastic variables, this comparison was made with the Welch t test, and finally, for the variables that did not follow a normal distribution, a nonparametric test (Mann-Whitney) was used. In all cases, a 95% confidence interval was used. Finally, for the study of the relationships between continuous variables, the Pearson correlation coefficient was calculated, as it has been considered the main statistic used to evaluate the marked objective, since it represents the correlation of the distances of the abutment with the opposing occlusal plane (mm) and the increases in stability (ISQ) according to RFA at the end of the follow-up period (8 weeks of osseointegration, T8).

In order to determine the sample size, a previous pilot study was performed. The main statistic (Pearson correlation coefficient), representing the correlation of the distance to the antagonist with the increase in RFA stability from T0 to T8, was 0.745 (*n* = 10 patients per group); therefore, assuming an alpha error of 5% and a test power of 80%, it was determined that a sample size of between 10 and 13 patients was sufficient. On the basis of these considerations, 15 patients were recruited for each group, for a total of 30 implants. Furthermore, there are studies, with resemblant methodology and objectives, published in the literature with similar sample sizes [22].

## 3. Results

A total of 30 patients undergoing unitary implants (*n* = 30) who met the established inclusion criteria were included in the sample. A total of four patients were excluded from the study because they did not attend the respective check-up: two of them in week 2 and another two in week 4. Additionally, two patients lost the preload of the healing abutment after week 2; therefore, they should also have been excluded because of the interference that this complication could cause in the osseointegration and stability of the implant. However, a new healing abutment was repositioned in each of these two implants, and although the patients were excluded from the study, their osseointegration occurred favorably. All these losses and missing data were replaced by new additions to the study to reach the final sample of 30 implants analyzed.

The characteristics of the population were an average age of 55 years (sd 10.63), of which 7 of the implants were placed in women (23.33%) and 23 in men (76.67%).

There were no osseointegration failures, and no additional procedural complications were reported in the final sample.

A total of 15 patients were included in each group: <4 and ≥4. In the <4 group, the range of distances was from 2.1 to 3.9 mm, with a mean of 3.15 (sd 0.574), and in the ≥4 group, the range of distances was from 4 to 7.7 mm, with a mean of 5.82 (sd 1.15), with a statistically significant difference between the groups (*p* < 0.05).

The implants in the sample (*n* = 30) classified by jaw where they were placed and the combination of diameters and lengths used are shown in Table 1. Twelve implants were placed in the upper jaw and 18 in the mandible, with 4 × 10 mm as the most widely used implant and 12 mm long implants as the least commonly used implants.

The average implant insertion torque was 31.33 Ncm (sd 5.16) for the <4 group and 28.76 Ncm (sd 5.81) for the ≥4 group, with no statistically significant differences between them (*p* = 0.16).

The descriptive and inferential statistics of the primary and secondary stability data measured by RFA for the two study groups are shown in Table 2. The differences in ISQ values between the groups were statistically analyzed for each implant by group and measurement time. The RFA data at T0, ΔT4–T2, and ΔT8–T4 were normal and homoscedastic. Similarly, the data for the variables ΔT8–T0, insertion torque, Δ bone mesial, and Δ bone distal were homoscedastic but did not follow a normal distribution. Finally, the RFA data at T8, although normally distributed, were not homoscedastic.

The difference in the secondary ISQ values between T8 and T0 is of special importance for inferring clinical conclusions since it represents the gain in stability as a result of the osseointegration process. In this regard, it can be verified that the <4 group showed an increase in ISQ values that was practically double that of the ≥4 group, with a statistically significant difference (*p* = 0.003912). However, it should be noted that at T0, the two groups had practically identical mean values, without statistically significant differences (*p* = 0.975).

Notably, the greatest increase between control sections occurred early (ΔT4–T2) for the implants in the <4 group, which suggests that the biomechanical stimulation of a higher- and closer-to-the-antagonist-tooth healing abutment promoted not only greater secondary stabilization but also faster or earlier stabilization, with statistically significant differences with respect to the ≥4 group (*p* = 0.0003106).

The descriptive and inferential statistics of the peri-implant marginal bone level, measured by parallelized radiography, and the differences in these levels between T8 and T0 for both groups, are presented in Table 3. In general, the means of bone loss at osseointegration were relatively low (range of −0.193–0.293), and no statistically significant differences in this variable were found between the groups.

**Figure 6:**
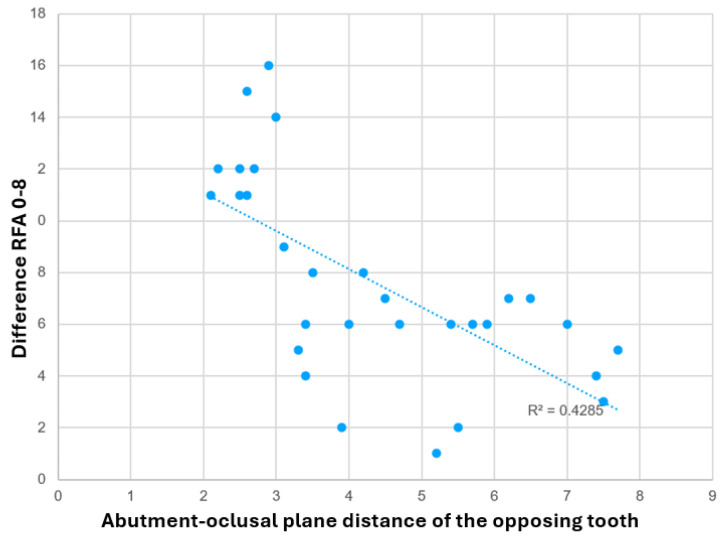
Scatter plot of the resonance frequency analysis (RFA) implant stability coefficient with respect to the healing abutment-occlusal plane distance of the opposing tooth.

Finally, when Pearson correlation analysis was performed between the individual values of separation (mm) of the coronal part of each healing abutment with the antagonist and the total increase in ISQ values that each implant achieved during the period of osseointegration (from T0 to T8), a negative correlation coefficient of −0.6546 was obtained, which implies a tendency towards a significant relationship with an average force and a linear inverse relationship, which implies that decreasing the distance between the abutment and the contact with the antagonist tends to increase the secondary ISQ values. The results are presented graphically in Figure 6.

## 4. Discussion

The main objective of this prospective observational study was to determine whether the implant healing abutment height and its proximity to the antagonist tooth influenced variables associated with the osseointegration of internal connection implants, such as implant stability measured through RFA and the evolution of bone proximal marginal levels analyzed by radiography.

The present study did not reveal differences between groups in the evolution of marginal bone levels during osseointegration but did reveal between-group differences in secondary stability values at the end of osseointegration and, additionally, in early phases of osseointegration, with superior results for the <4 mm group.

However, it must be recognized that the method of measurement of both variables does not have the same level of precision. Although parallelized radiography is a commonly used method for measuring marginal bone levels in the literature [23,24], the likelihood of error is higher with this method, since, although it is carried out by digital tools, the selection of the point of reference depends on the surgeon. In our study, we attempted to limit this error with a reproducibility test, which was performed by a second surgeon, and an adequate between-surgeon concordance index was observed. RFA with Penguin^®^ is a biometric test with adequate repeatability and reproducibility, but it also allows measurements to be carried out throughout the osseointegration process, monitoring its evolution and revealing a strong relationship between the evolution of bone stiffness and micromotion at the bone-implant interface [25,26,27].

Additionally, it should be noted that the design of the present study was observational, and patients were not randomly assigned to groups. Although this design could constitute a bias, when the primary stability data of the implants in both groups were analyzed, there were no statistically significant differences in the insertion torque, ISQ values (RFA), or practically identical means (70.67 in the <4 mm group and 70.73 in the ≥4 mm group).

These results are similar to the values obtained in other studies, both in animal and clinical models. An example in this regard is the work of Diéguez Pereira et al. placing dental implants in rabbit tibias, with mean ISQ values of 67 ± 6.1, or the clinical study of Rodrigo et al., with a sample size of 4114 implants placed in 1680 patients, with a mean SIS of 73.96 ± 6.28 [28,29].

During the osseointegration period, the stability values of all implants in the study increased, thus reflecting the exchange of primary mechanical stability for secondary biological stability. This increase in ISQ values occurs because, during osseointegration, the progressive apposition of the bone matrix on the implant surface and its mineralization leads to an increase in the rigidity of the bone union in the implant, which decreases implant micromotion capability. This circumstance is easily detectable via RFA. This phenomenon was described by Meredith in 1996 through a simple in vitro study with implants placed in self-curing resin. The study revealed that as the resin polymerized and therefore rigidity increased, the ISQ values of the implants increased [25,30,31,32].

The present study revealed greater increases in the stability of implants in the group with the smallest interocclusal space (<4 mm group) than in those of the group with the largest space (≥4 group), with statistically significant differences at the end of the osseointegration process (T8). This fact highlights the possible influence of the biomechanical stimulus that a greater healing abutment may have on the biological stability of the implants. The results are in line with those of immediate loading studies, both in animal models [19] and in clinical observations [1]. Similar results were obtained by Aklogan et al. In a prospective clinical study with 39 unitary implants in the posterior maxilla, the implants in which immediate or early loading was performed achieved significantly higher ISQ values during the osseointegration process than those in which a conventional load was applied [22].

In addition, the study revealed not only that the ISQ values were significantly greater in the <4 mm group than in the ≥4 group but also that the greatest increase in ISQ values occurred earlier, with a distinguishable peak occurring between T2 and T4. Indeed, during this short period of 2 weeks, the implants in the group with a space smaller than 4 mm obtained an average increase in the ISQ of 6.8, whereas in the group with a space greater than or equal to 4 mm, the average increase in the ISQ was 3.1. Therefore, it seems that the biomechanical effect of high healing abutments not only results in higher ISQ values but also in earlier osseointegration, which may be of special clinical interest in situations where acceleration of the process is needed.

This finding is reminiscent of a similar effect that occurs with implants with bioactive surface modifications, in which the achievement of adequate BIC and increased ISQ values occurs in the first 4 weeks of integration instead of the 8 weeks characteristic of conventional surfaces. In this context, an observational clinical study [33] revealed the possibility of integration and stabilization in an early loading protocol (4 weeks) when surface-treated implants with one of these improved surfaces were used. Likewise, this stabilization and accelerated integration has also been observed in studies evaluating the use of hyperelastic alloys in the manufacture of implants, in this case, using histological criteria. Importantly, while the effect described, both in the case of our study and in the case of hyperelastic alloys, may have a biomechanical cause, in the specific case of bioactive surfaces, this effect is due to other cellular and metabolic mechanisms that promote these surfaces. However, all the implants used in the study (Essential Cone Klockner; Soadco, Escaldes-Engordany, Andorra) consisted of a conventional surface modification SLA (blasting of alumina particles and acid attack); therefore, we did not introduce a covariate or a confounding bias in this regard [34].

Although this study provides useful information for clinical application, it is not without its limitations. One limitation may be that RFA was performed on the healing abutment instead of in the conventional way on the implant connection itself. There is literature that supports that performing measurements through these healing abutments does not influence the results compared with performing them directly on the implant, and it is evident that, by always doing so on the abutments, the comparison between measurements represents the evolution without confusion [35].

On the other hand, the RFA methodology for the abutment may entail an additional advantage: the fact that it is not necessary to release the abutment from the connection for the assessment of stability in clinical practice, which may lead to a decrease in the number of the successive disconnections and connections of the abutments, thereby limiting the possible complications that may arise from this practice. We have not found evidence in this regard in the literature, but with regard to the technique of ‘one abutment one time’ in prosthetic intermediate abutments, multiple disconnections significantly affect marginal bone loss in comparison with the placement of the definitive abutment on the day of surgery [36,37].

Other limitations include those inherent to the difficulties of radiographically measuring marginal bone levels, as discussed above. However, it is not easy to determine whether the observable effect on the results would be replicable in bone-level implants instead of implants with a juxtaposed polished neck, as used in the present study. Regarding bone-level implants, the abutment connection is at the bone or even infra-bony level, and perhaps micromotion resulting from mechanical stimulation from a higher abutment at that level may have an undesired effect. Finally, despite being able to verify the differences in stability using RFA, the results do not allow us to draw conclusions on histological improvements, which should be evaluated in animal study models.

Finally, the use of different combinations of implant lengths and diameters in this study may be a limitation. However, more than half of the implants used were 4 × 10 mm long. In addition, several studies have revealed that the diameter and length of the implant do not usually lead to significant differences in the stability values measured by RFA [28].

## 5. Conclusions

This study revealed that the use of higher healing abutments, whose coronal platform is at a distance of less than 4 mm with respect to the occlusal plane of the opposing piece, is of interest for achieving higher secondary stability values and even earlier stability.

Regarding the variable of peri-implant bone level, no differences were observed between groups in which the distance was less than 4 mm and those in which the distance was greater than 4 mm.

## Figures and Tables

**Table 1:** Number of implants used in the sample, classified according to the jaw in which there were placed and the combination of diameter and length used (mm).

	4 × 8 mm Implant	4 × 10 mm Implant	4 × 12 mm Implant	4.5 × 8 mm Implant	4.5 × 10 mm Implant	4.5 × 12 mm Implant
Upper Jaw	3	6	0	2	1	0
Jaw	5	10	0	3	0	0

**Table 2:** Descriptive and inferential statistics of the stability values measured by resonance frequency analysis (RFA) (implant stability quotient (ISQ)), with between-group comparisons according to the time points of the measurement of the variable. * Statistically significant.

Group	Time Δ RFA	RFA (ISQ) n	Medium	sd	Median	Statistical Test	*p* Value
<4	T0	15	70.67	4.35	71	Student’s *t*	0.975
≥4		15	70.73	6.930	69		
<4	ΔT2–T4	15	6.800	2.704	7	Student’s *t*	0.0003106 *
≥4		15	3.133	2.100	3		
<4	ΔT8–T4	15	5.067	2.987	5	Student’s *t*	0.167
≥4		15	3.733	2.052	3		
<4	ΔT0–T8	15	9.870	4.120	11	Mann-Whitney	0.003912 *
≥4		15	5.333	1.988	6		
<4	T8	15	82.000	4.040	82	Weich’s *t*	0.01148 *
≥4		15	76.070	7.280	74		

**Table 3:** Descriptive and inferential statistics of the differences in the marginal, mesial and distal bone levels between T8 and T0 for both groups under study.

Δ T8–T0 Radiographic Proximal Marginal Bone Levels (mm)							
Group	Location	n	Medium	sd	Median	Statistical Test	*p* Value
<4	Mesial	15	−0.240	0.188	−0.200	Mann-Whitney	0.3094
≥4		15	−0.193	0.246	−0.100		
<4	Distal	15	−0.280	0.237	−0.200	Mann-Whitney	0.8167
≥4		15	−0.293	0.202	−0.300		

## Data Availability

The data that support the findings of this study are not openly available for reasons of sensitivity but are available from the corresponding author upon reasonable request.

## Abbreviations

RFA	Resonance Frequency Analysis
ISQ	Implant Stability Quotient
BIC	Bone-to-Implant Contact
Ncm	Newton
FDI	World Dental Federation
ASA	American Society of Anesthesiologists
